# C6/36 *Aedes albopictus* Cells Have a Dysfunctional Antiviral RNA Interference Response

**DOI:** 10.1371/journal.pntd.0000856

**Published:** 2010-10-26

**Authors:** Doug E. Brackney, Jaclyn C. Scott, Fumihiko Sagawa, Jimmy E. Woodward, Neil A. Miller, Faye D. Schilkey, Joann Mudge, Jeffrey Wilusz, Ken E. Olson, Carol D. Blair, Gregory D. Ebel

**Affiliations:** 1 Department of Pathology, University of New Mexico School of Medicine, Albuquerque, New Mexico, United States of America; 2 Arthropod-Borne and Infectious Diseases Laboratory, Department of Microbiology, Immunology and Pathology, Colorado State University, Fort Collins, Colorado, United States of America; 3 National Center for Genome Resources, Santa Fe, New Mexico, United States of America; The University of Queensland, Australia

## Abstract

Mosquitoes rely on RNA interference (RNAi) as their primary defense against viral infections. To this end, the combination of RNAi and invertebrate cell culture systems has become an invaluable tool in studying virus-vector interactions. Nevertheless, a recent study failed to detect an active RNAi response to West Nile virus (WNV) infection in C6/36 (*Aedes albopictus*) cells, a mosquito cell line frequently used to study arthropod-borne viruses (arboviruses). Therefore, we sought to determine if WNV actively evades the host's RNAi response or if C6/36 cells have a dysfunctional RNAi pathway. C6/36 and *Drosophila melanogaster* S2 cells were infected with WNV (*Flaviviridae*), Sindbis virus (SINV, *Togaviridae*) and La Crosse virus (LACV, *Bunyaviridae*) and total RNA recovered from cell lysates. Small RNA (sRNA) libraries were constructed and subjected to high-throughput sequencing. In S2 cells, virus-derived small interfering RNAs (viRNAs) from all three viruses were predominantly 21 nt in length, a hallmark of the RNAi pathway. However, in C6/36 cells, viRNAs were primarily 17 nt in length from WNV infected cells and 26–27 nt in length in SINV and LACV infected cells. Furthermore, the origin (positive or negative viral strand) and distribution (position along viral genome) of S2 cell generated viRNA populations was consistent with previously published studies, but the profile of sRNAs isolated from C6/36 cells was altered. In total, these results suggest that C6/36 cells lack a functional antiviral RNAi response. These findings are analogous to the type-I interferon deficiency described in Vero (African green monkey kidney) cells and suggest that C6/36 cells may fail to accurately model mosquito-arbovirus interactions at the molecular level.

## Author Summary

Cell culture systems are invaluable tools for studying virus-host interactions. These systems are typically easy to maintain and manipulate; however, they can fail to accurately mimic the host environment encountered by viruses. Therefore, defining the limitations of each system is critical to properly interpreting the results. C6/36 *Aedes albopictus* cells are commonly used to study arthropod-borne viruses (arboviruses), such as West Nile virus (WNV). Recent evidence suggests that the RNA interference (RNAi) pathway, a critical aspect of the cellular innate antiviral immune response in invertebrates, may not actively target WNV in C6/36 cells. However, it is unknown whether this observation is limited to WNV. Therefore, we examined small RNA populations from C6/36 and *Drosophila melanogastor* S2 cells infected with WNV, Sindbis virus and La Crosse virus by high-throughput sequencing. We demonstrate that the RNAi pathway actively targets each of the three viruses in S2 cells, but does not in C6/36 cells. These findings suggest that C6/36 cells may fail to accurately model mosquito-arbovirus interactions.

## Introduction

RNA interference (RNAi) is a process by which intracellular long double stranded RNA (dsRNA) is cleaved into small RNA (sRNA) effector molecules that direct the silencing of complementary RNA sequences. Multiple pathways, including exo- and endo-small interfering RNA (siRNA), microRNA (miRNA) and PIWI-interacting RNA (piRNA), contribute to this process. The exo-siRNA pathway, in which silencing is triggered by exogenously derived dsRNA molecules, is thought to comprise the main antiviral response in mosquitoes [1]–[7]. This pathway is initiated when Dicer 2 (Dcr2) binds to and cleaves long dsRNA molecules that exist within cells as viral replicative intermediates and/or RNA secondary structures into 20–22 nucleotide siRNAs [8]–[11]. The resulting siRNAs are loaded by Dcr2 and R2D2 into the multi-protein RNA-induced silencing complex (RISC), which includes Argonaut 2 (Ago2), and unwound, after which the 3′ terminus of the retained guide strand is 2′-O-methylated [12], [13]. The siRNA-loaded RISC identifies single stranded RNAs complementary to the guide strand, which are cleaved by the endoribonucleolytic activity of Ago2 [14], [15]. The advent of high-throughput sequencing technologies allows researchers to characterize viral-derived siRNAs (viRNAs) and to quantitatively map the areas of the viral genome most often targeted [1], [5], [6], [16], [17].

Arthropod-borne viruses (arboviruses) are a diverse group of viruses maintained in nature by horizontal transmission between hematophagous, arthropod vectors and vertebrates. Arbovirus infections can cause an acute, pathogenic outcome in the vertebrate host, but establish a persistent, relatively non-pathogenic infection in the invertebrate vector, with some noted exceptions [18]–[20]. The reasons for this difference are not fully understood, but may be related to the highly inflammatory innate antiviral immune response in vertebrates (type-I interferon-mediated) compared to invertebrates, which rely on antiviral mechanisms such as RNAi and the Toll, JAK/STAT, and Imd/Jnk signaling pathways [7], [18], [21]–[23]. Of these, RNAi appears to be the primary means of limiting viral infections in the vector [2]–[4], [7], [16], [24]–[27]. High-throughput sequencing has identified viRNAs from *Aedes aegypti* and *Culex pipiens quinquefasciatus* mosquitoes infected with Sindbis virus (SINV, *Togaviridae*: *Alphavirus*) and West Nile virus (WNV, *Flaviviridae*: *Flavivirus*), respectively, and *Drosophila* infected with Flock House virus (FHV, *Nodaviridae*: *Alphanodavirus*) [1], [6], [16]. In each case, the viRNAs were predominantly 21 nt in length, asymmetrically distributed across the length of the genome and derived from both the positive and negative RNA strands.

*Aedes albopictus* C6/36 and *Drosophila melanogaster* S2 cells are commonly used, immortalized, invertebrate cell lines that have become powerful and convenient tools for studying many host/virus interactions at the molecular level. Originally established from mosquito larvae homogenates, C6/36 cells are easy to maintain and highly permissive to numerous arboviruses. Likewise, S2 cells are easy to maintain and manipulate and critical specific reagents, such as antibodies and specific knockouts, are available commercially. However, immortalized cells may not accurately model the natural environment encountered by viruses in the whole organism. For example, Vero (African green monkey kidney) cells, which are used to study many human viruses because of their inherent permissiveness, lack a functional type-I interferon response [28]. A recent report on WNV infection in insect cell culture models raised the possibility that C6/36 cells may be similarly deficient in key components of their antiviral defense mechanisms, leading to their permissiveness for arbovirus infection [29]. Therefore, we investigated the RNAi response in C6/36 cells compared to S2 cells. Specifically, C6/36 and S2 cells were infected with representatives of three diverse arbovirus families and small RNA populations of infected cells were characterized by deep sequencing.

## Materials and Methods

### Viruses

The viruses used in these studies are representative of each of three major arbovirus families that include many human pathogens; *Flaviviridae*, *Togaviridae*, and *Bunyaviridae*. WNV (*Flaviviridae*; *Flavivirus*) was generated from an infectious cDNA clone derived from the NY99 strain [30]. SINV (*Togaviridae*, *Alphavirus*) was generated from the SINV TE3′2J infectious clone [31]. The LACV (*Bunyaviridae*; *Orthobunyavirus*) used in these studies was the LACV/Human/1960 strain (GenBank accession nos. EF485032.1, EF485031.1, and EF485030.1). Originally, isolated from the brain of a LaCrosse encephalitis patient in 1965 the virus was subsequently passaged in suckling mice three times and baby hamster kidney cells (BHK-21) an additional six times. Stock virus was prepared in BHK-21 cells [32].

### Cell Culture, Virus Infections and RNA Extractions

The C6/36 *Aedes albopictus* cells were grown in Dulbecco's modified essential medium (DMEM) with 10% fetal bovine serum (FBS), 100 U/ml penicillin, 100 µg/ml streptomycin, L-glutamine, and sodium bicarbonate at 28°C with CO_2_. The S2 *Drosophila melanogaster* cells were grown in Schneider's *Drosophila* medium with 10% FBS, penicillin/streptomycin, and L-glutamine at 28°C without CO_2_.

C6/36 and S2 cell cultures were infected in triplicate with each of the three viruses at a multiplicity of infection (MOI) of 0.1 and 1, respectively. Considering that *Drosophila* is not a natural host for any of these viruses, higher MOIs were required to ensure infection. Virus stocks were diluted in maintenance medium with a FBS concentration of 2%, inoculated onto confluent cell monolayers, and allowed to adsorb for one hour at room temperature. The virus inocula were removed and replaced with maintenance medium. Five days post infection, WNV and SINV infected C6/36 cells were harvested. LACV infection of C6/36 cells and all infections in S2 cells were maintained an additional two days before harvesting. Cells were pelleted by centrifugation and resuspended in mirVana lysis buffer. RNA from each sample was extracted using the mirVana miRNA Isolation Kit (Ambion, Austin, TX) according to the manufacturer's instructions. RNA quantity and integrity was determined on an Agilent 2100 Bioanalyzer (Agilent Technologies, Santa Clara, CA).

### Preparation of Small RNA Libraries and High-Throughput Sequencing

Equal amounts of RNA from the three replicates were pooled and ethanol precipitated. Approximately 10 µg of total RNA from each experimental group was size fractionated on a TBE/urea 15% polyacrylamide gel and small RNA (sRNA) populations (17–30 nt) recovered. 5′ and 3′ sequencing adapters (5′ adapter: 5′-GUUCAGAGUUCUACAGUCCGACGAUC-3′, 3′ adapter: 5′-P-UCGUAUGCCGUCUUCUGCUUGU-3′) (Oligonucleotide sequences © 2007-2009 Illumina, Inc. All rights reserved) were then ligated to sRNAs using T4 RNA ligase, of which the 3′ adapter was not pre-adenylated. The sRNAs were reverse transcribed and PCR amplified according to the manufacturer's instructions (Illumina, San Diego, CA). The resulting libraries were sequenced at the National Center for Genome Resources (Santa Fe, NM) using an Illumina Cluster Station and Genome Analyzer II or IIx.

### Assembly and Analysis of sRNA Libraries

Reads from sRNA libraries were trimmed of adapter sequences and aligned to genome sequences of either the WNV NY99 infectious clone, the SINV TE3′2J infectious clone or the LACV/Human/1960 strain using the Short Oligonucleotide Alignment Package v.1 (SOAP) (http://soap.genomics.org.cn/). A seed size of eight and a maximum of two mismatches were permitted. Gaps and further trimming were not allowed. Quality scores represent an average of the confidence in each sequenced nucleotide in each sRNA. These are based on the Illumina scoring system where 1 is a minimum and 40 is the maximum. Additional analyses were performed using Microsoft Excel and GraphPad.

## Results

### Characterization of Small RNA Profiles

**Figure 1:**
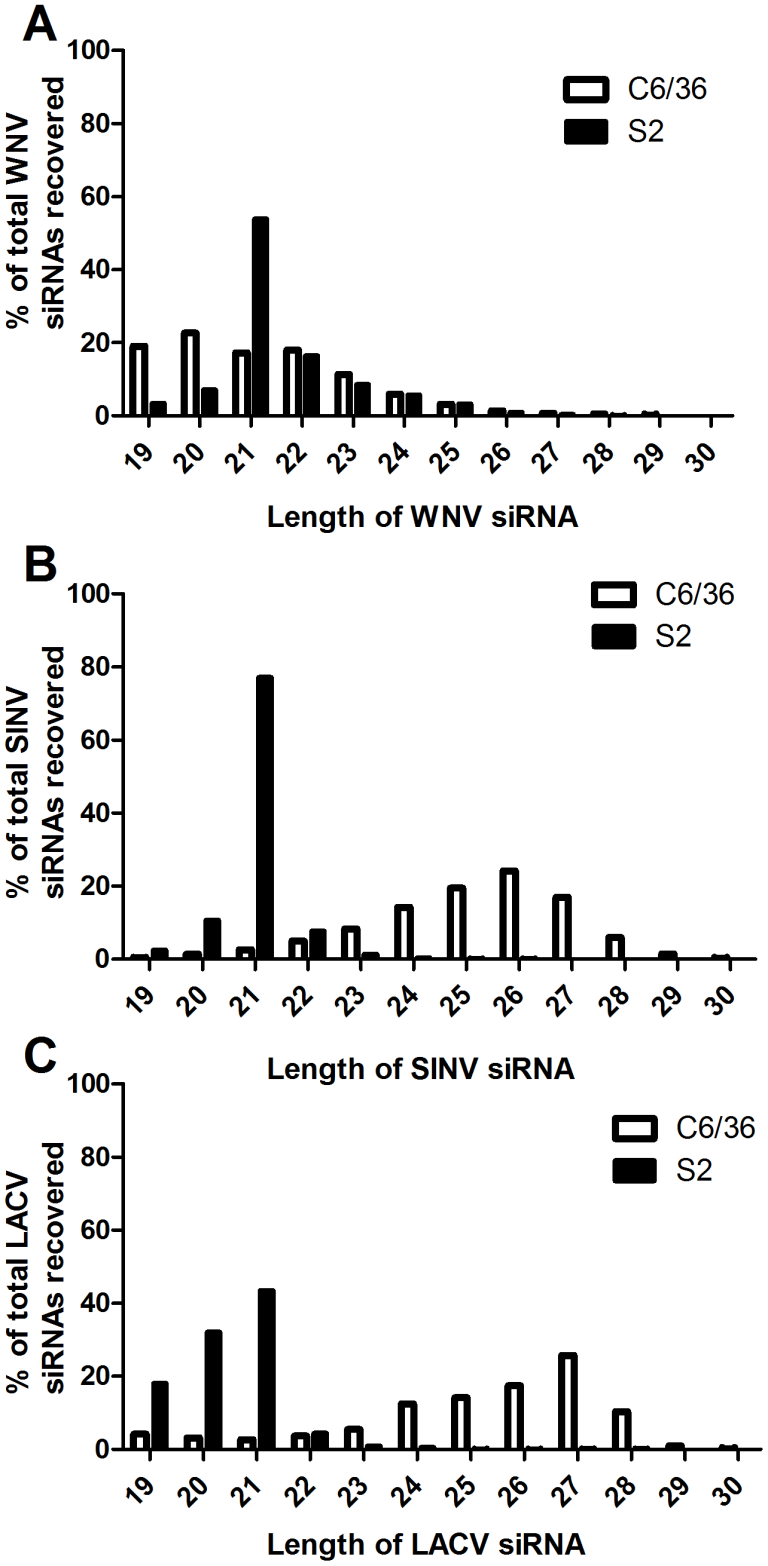
Size and abundance of small RNA reads Mmapping to the viral genomes. The abundance of 19–30-mer sRNA reads mapping to the WNV (A), SINV (B) and LACV (C) genomes based on size. Abundance is represented as a percentage of the total viRNAs from each sample. The black bars correspond with samples collected from S2 cells and white bars from C6/36 cells.

To assess the antiviral RNAi response in two model invertebrate cell culture systems, we sequenced small RNAs (sRNAs) from C6/36 and S2 cells infected with three arboviruses, WNV, SINV and LACV (*Bunyaviridae: Orthobunyavirus*). These viruses were chosen as representative members of three major arbovirus families with both positive- (WNV, SINV) and negative-polarity (LACV) RNA genomes. The total number of sRNA reads obtained varied greatly between samples and most likely reflects differences in cell density at the experimental endpoint, since the majority of the sRNAs originate from the host in the form of miRNAs. In WNV infected cells, there were 3.4 and 9.9 million reads from C6/36 and S2 cells, respectively (Table 1). Of these, only 12,539 reads (0.37%; 6,151 unique) in C6/36 cells and 4,431 (0.045%; 3,127 unique) in S2 cells aligned to the WNV genome, where unique reads represent individual viRNAs that code for a specific nucleotide sequence. In both cell lines, greater than 87% of the reads perfectly aligned with the WNV genome. Analysis of the viRNA sizes revealed that over 76% of the viRNAs isolated from S2 cells were 20–22-mers (mean length 21.6 nt) with 54% constituting 21-mers. In contrast, 57% of the viRNAs from C6/36 cells were 20–22-mers (mean length 21.3 nt) and 17.2% were 21-mers (Figure 1A) with a high proportion of the WNV viRNAs being 17–18 mers (data not shown).

**Table 1:** Small RNA profiles from C6/36 and S2 infected cells.

		Reads Aligning To Viral Genome			Reads With Mismatches n (%)		
Small RNA Library	Total # of Reads (×10^6^)	Total # (unique #)	Average Length	Average Quality Score	0	1	2
WNV C6/36	3.4	12,539 (6,151)	21.3	39.8	11,399 (91)	964 (8)	176 (1)
WNV S2	9.9	4,431 (3,127)	21.6	29.4	3,912 (88)	363 (8)	156 (4)
SINV C6/36	14.4	1.6×10^6^ (1.2×10^5^)	25.2	30.8	1.5×10^6^ (92)	1.1×10^5^ (7)	19,411 (1)
SINV S2	7.3	3.5×10^5^ (46,103)	21.0	31.4	3.1×10^5^ (91)	27,070 (8)	4,202 (1)
LACV C6/36	11.2	9.4×10^5^ (73,098)	25.2	31.2	8.7×10^5^ (92)	65,265 (7)	9,672 (1)
LACV S2	8.6	6,777 (2,201)	20.4	29.2	5,899 (87)	683 (10)	195 (3)

Analysis of the sRNA populations in SINV infected cells revealed that there were 14.4 and 7.3 million reads in C6/36 and S2 cells, respectively (Table 1). Of these, 1.6×10^6^ reads (11.11%; 1.2×10^5^ unique) in C6/36 and 3.5×10^5^ reads (4.79%; 46,103 unique) in S2 cells aligned to the SINV genome, of which greater than 90% perfectly aligned. As with the WNV infected cells, greater than 93% of the viRNAs isolated from S2 were 20–22-mers (mean length 21 nt) and 76% were 21-mers (Figure 1B). In C6/36 cells, only 9% of SINV viRNAs were 20-22 nt in length. Mean length was 25.2 nt, with the majority of the viRNAs (76%) being between 24–27 nt.

Finally, there were 11.2 and 8.6 million reads in LACV infected C6/36 and S2 cells, respectively (Table 1). In LACV infected C6/36 cells, 9.4×10^5^ reads (8.39%; 73,098 unique) aligned with the LACV genome, with greater than 90% of the reads having zero mismatches. In contrast to the C6/36 infected cells, only 6,777 reads (0.078%; 2,201 unique) from S2 cells aligned with the LACV genome; however, 87% of these reads matched perfectly to LACV RNA. Consistent with the results from WNV and SINV, the majority of the LACV viRNAs in S2 cells were 20–22-mers (80%), with 43% of these reads being 21 nt in length (mean length 20.4 nt) (Figure 1C). In C6/36 cells, only 9% of the total viRNAs were 20–22-mers. Mean length of LACV viRNAs from C6/36 cells was 25.2 nt, with the majority (79%) being between 24–28 nt in length.

### Distribution and Abundance of viRNAs

**Figure 2:**
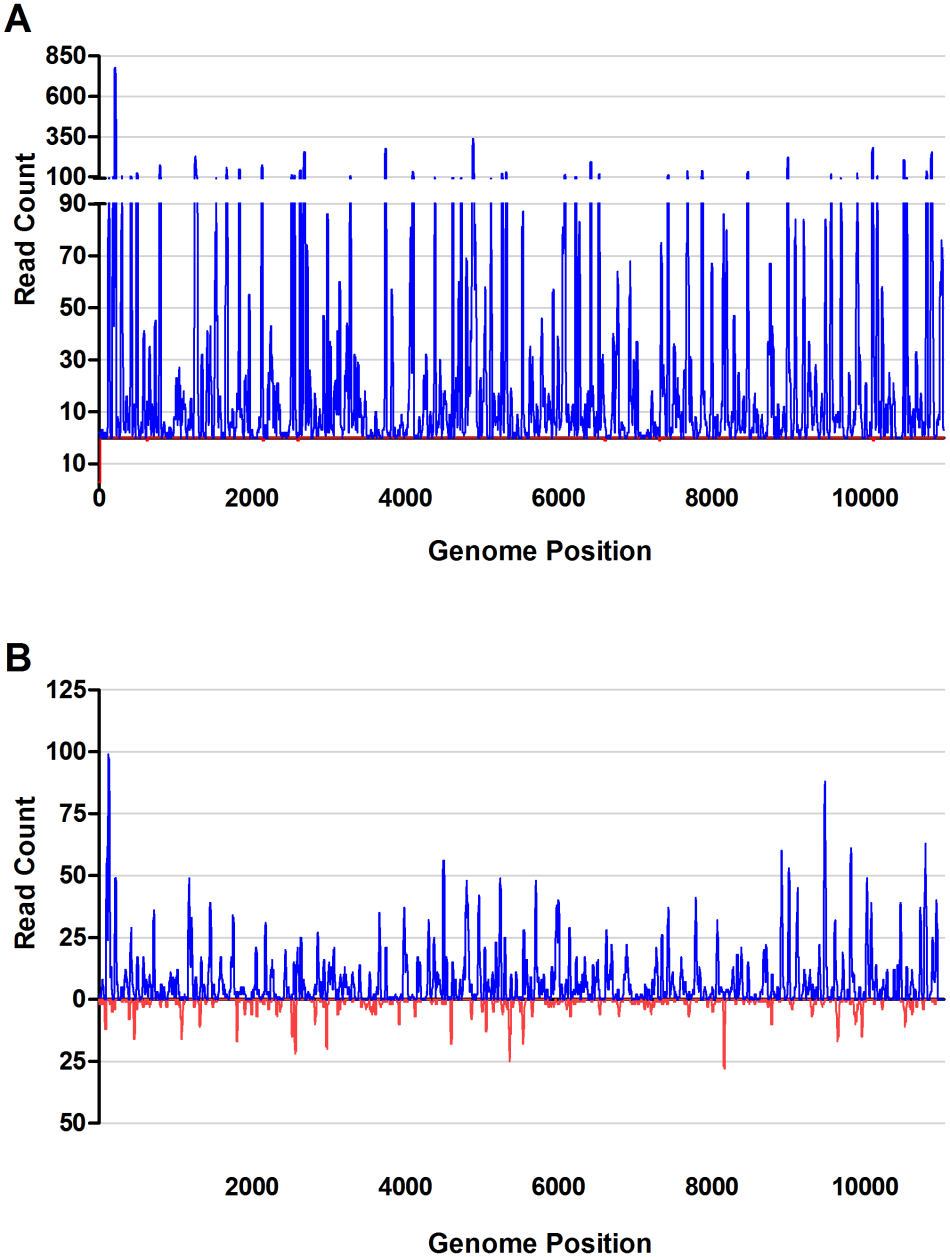
viRNA coverage of the WNV genome in C6/36 and S2 cells. Complete genome of WNV (11,029 nt.) showing intensity at each nucleotide of the genome in C6/36 (A) and S2 (B) cells. Plotted are the 19–30-mer viRNA reads. Reads originating from the genomic, positive strand are represented in blue above the x-axis and those originating from the negative strand are represented in red below the x-axis.

To more closely examine the viRNA populations, viRNAs from each experimental sample were aligned to the input viral genome and viRNA coverage intensity determined per nucleotide across the length of the genome. Included in these analyses are all viRNA reads 19–30 nucleotides in length. Over 90% of the WNV genome was targeted by at least one viRNA in both C6/36 and S2 cells. Inspection of the intensity of viRNA coverage of the WNV genome revealed significant positional and regional differences between C6/36 and S2 cells (Figure 2). viRNAs isolated from infected C6/36 cells were asymmetrically distributed across the length of the genome and were derived almost exclusively from the positive sense viral genomic RNA strand (vRNA) (99.9%) (Figure 2A). The most highly targeted site was genome position 206 within the capsid coding sequence with 776 reads covering this position. Expansion of the data set to include 17–18-mers revealed that the first 17 nucleotides of the complementary, negative RNA strand were the most highly targeted (1,019 reads mapping to these sites) within the genome (data not shown). Alignment of viRNAs obtained from WNV infected S2 cells revealed that they were asymmetrically distributed and were derived from both the positive sense vRNA (84%) and complementary cRNA (16%) (Figure 2B). Nucleotide 125 located in the capsid coding sequence was the most targeted site within the genome (99 reads mapping to this site).These results are strikingly similar to those generated from WNV infected *Cx. p. quinquefasciatus* midguts [1].

**Figure 3:**
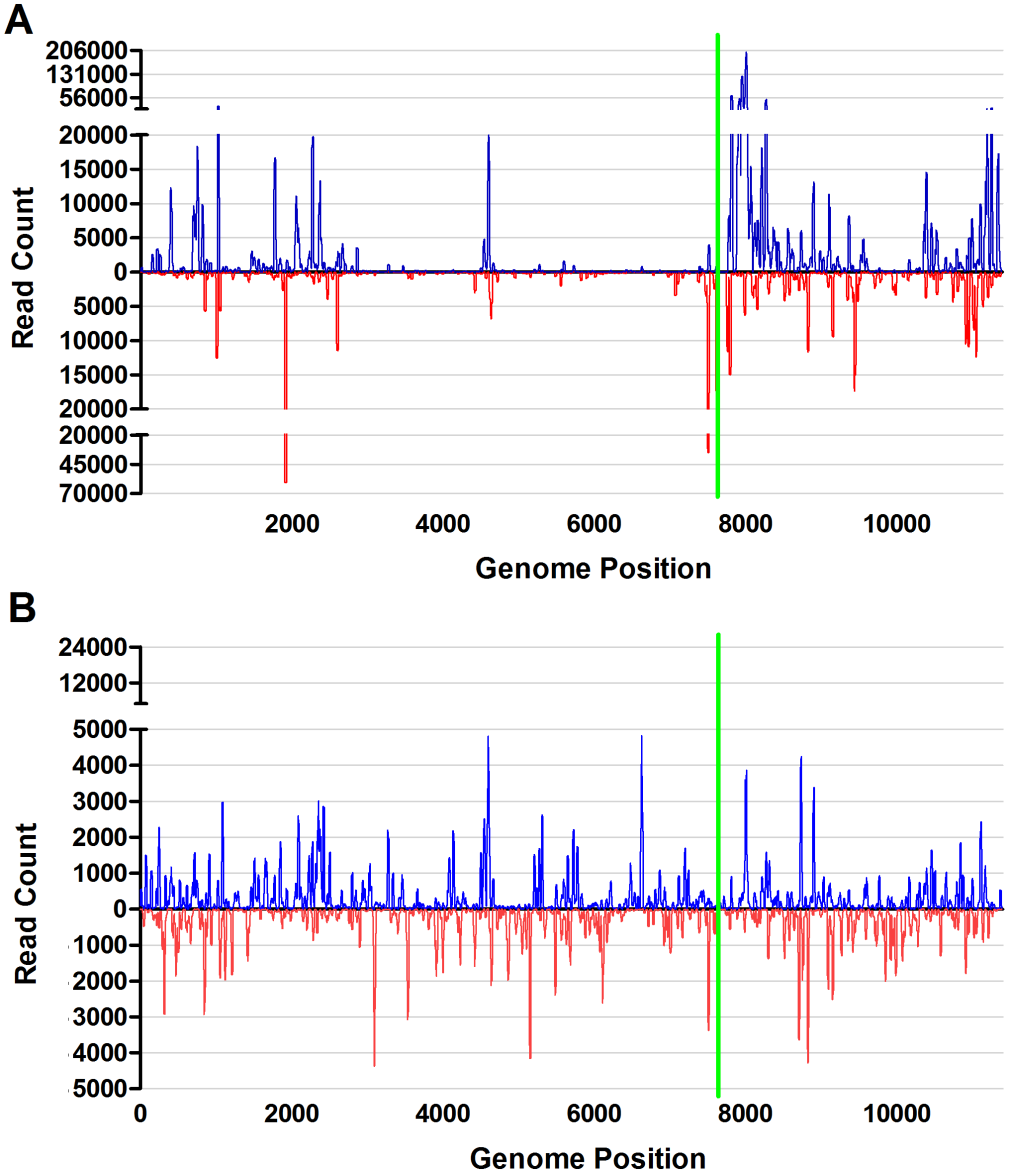
viRNA coverage of the TE3′2J SINV genome in C6/36 and S2 cells. Complete genome of TE3′2J SINV (11,385 nt.) showing intensity at each nucleotide of the genome in C6/36 (A) and S2 (B) cells. Plotted are the 19–30-mer viRNA reads. Reads originating from the genomic, positive strand are represented in blue above the x-axis and those originating from the negative strand are represented in red below the x-axis. The green vertical line represents the location of the subgenomic promoter.

The viral genome coverage, viRNA frequency and distribution for the SINV infected C6/36 and S2 cells were very similar to those observed for WNV infected cells. In both cell lines 100% of the viral genome was targeted by at least one viRNA. However, the percent coverage in the C6/36 cells is deceiving as the majority of viRNAs were directed at only a few “hotspots”. The viRNAs from C6/36 cells were unevenly distributed across the genome with both positive sense vRNA (71%) and negative sense cRNA (29%) targeting (Figure 3A). The region just 3′ of the SINV subgenomic promoter was the most highly targeted with 201,104 reads mapping to nucleotide 8,013, located in the viral capsid coding region. The increased targeting of the subgenomic transcripts may reflect the overall abundance of these transcripts in comparison to the full length genome. This predilection for targeting the subgenomic transcript was not observed in the SINV infected S2 cells (Figure 3B) or SINV infected *Ae. aegypti* [6]. Further similarities between the S2 cell viRNA profile and that from *Ae. aegypti* included the asymmetry of distribution across the genome and the proportion of viRNAs derived from the positive sense vRNA (52% in S2 cells and 54% in *Ae. aegypti*) [6]. The most highly targeted site within the viral genome was nucleotide 4,601 (4,803 reads) in the viral nsp3 gene.

**Figure 4:**
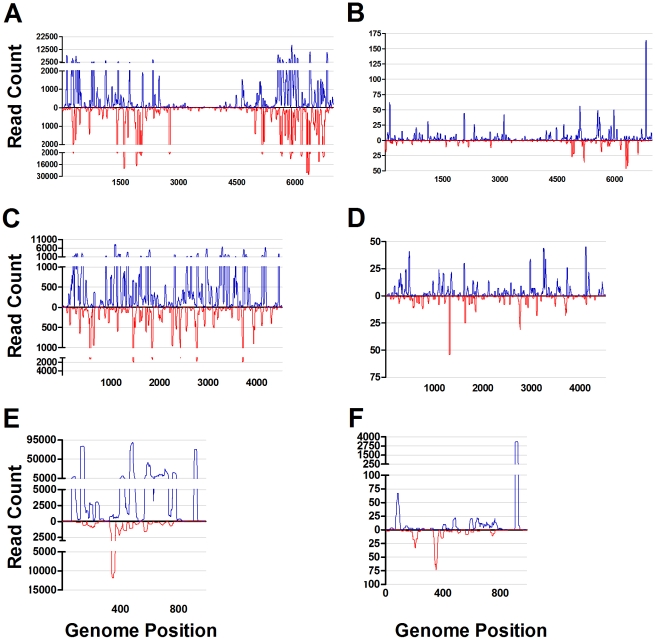
viRNA coverage of the LACV/Human/1960 strain genome in C6/36 and S2 cells. Complete genome of LACV/Human/1960 strain showing intensity at each nucleotide of the genome in C6/36 (A,C,E) and S2 (B,D,F) cells. A and B correspond with the L gene segment (6,980 nt), C and D the M gene segment (4,526 nt), and E and F to the S gene segment (984 nt). Plotted are the 19–30-mer viRNA reads across the length of each segment represented by the x-axis. Reads originating from the genomic, negative strand are represented in red below the x-axis and those originating from the positive strand are represented in blue above the x-axis.

Analysis of sRNAs revealed 100% viRNA coverage of the LACV genome in both C6/36 and S2 cells. viRNAs from both cell lines were asymmetrically distributed across the genome and were derived from both the vRNA (negative-sense) and cRNA (positive-sense) strands (Figure 4). The predominance of positive-polarity strand targeting observed for WNV and SINV in C6/36 cells was also observed in both cell types infected with the negative-sense RNA LACV. However, a difference in the proportions of negative- and positive-sense targeting was noted. In S2 cells, 84% of the viRNAs were derived from the cRNA (positive-sense) strand compared to 72.5% in C6/36 cells (Figure 4B, D, F and 4A, C, E, respectively). The intensity of viRNA targeting for each of the three segments of the tri-partite LACV genome (L segment 6,980 nt, M segment 4,526 nt and S segment 984 nt) was determined. Values are presented as the total number of viRNAs per kb per segment. The S segment was the most frequently targeted in both cell types with 4.3×10^5^ and 3,829 viRNA reads per kb in C6/36 and S2 cells, respectively. As expected, the most frequently targeted sites within the genome were located in the S segment. The predominant S segment region targeted in LACV infected S2 cells was located between nt 904-923 at the 3′ end (∼3,500 hits), while nt 482 (89,871 hits) was the most targeted site in C6/36 cells (Figures 4E and 4F). In each cell line there was an approximately 10-fold reduction in the targeting of the M and L segments as compared to the S segment (M = 3.0×10^4^ and L = 5.5×10^4^ in C6/36 cells and M = 263 and L = 261 in S2 cells) (Figure 4A–D). The observed S segment targeting bias most likely reflects the abundance of S segment mRNA in comparison to M and L segment mRNA in bunyavirus-infected cells [33].

## Discussion

Cell culture systems have become invaluable tools in the study of host-virus interactions. However, they may not faithfully model certain molecular features of the host organism-virus interaction. As a result, interpreting data generated in these systems can have limitations and clearly defining the limitations of such systems is crucial to glean as much accurate information as possible from these studies. C6/36 *Aedes albopictus* cells are a mosquito cell line commonly used to study arbovirus-vector interactions [34]–[36]. Recently, it was demonstrated that WNV-specific siRNAs could not be detected by northern blot hybridization following infection of C6/36 cells, although siRNAs were found in *Drosophila* S2 cells [29]. The authors concluded that WNV actively evaded the antiviral RNAi response either through the activity of a, as yet unidentified, WNV encoded viral suppressor of RNAi or by sequestration of viral replicative complexes within protective membranous vesicles [37], [38]. However, it remains unclear why these observations were limited to C6/36, but not S2 cells. Therefore, we tested the hypothesis that C6/36 cells lack a fully functional antiviral RNAi response. To this end, C6/36 and S2 cells were infected with members of three taxonomically diverse arbovirus families and the RNAi response characterized by high-throughput sequencing of viRNAs. Examination of three unrelated arboviruses allowed us to determine if the observed results were specific to a particular virus and its associated strategies for evading host defense, or a general defect in the cells themselves.

We prepared six sRNA libraries from WNV, SINV and LACV infected C6/36 and S2 cells. In general, the total number of sequence reads and the average base call quality scores were comparable, with the exception of the WNV infected C6/36 cell library, which had fewer total reads and higher quality scores (Table 1). This observed difference may be attributable to the library preparation or sequencing efficiency, as the WNV infected C6/36 cell library was prepared and sequenced independently of the other samples. Nevertheless, the results (average read lengths and sRNA reads perfectly aligning to the viral genomes) were consistent among samples and with previous studies and therefore provide confidence that informative comparisons can be made between libraries. Inspection of sRNA sequencing results from infected C6/36 and S2 cells revealed obvious differences between the proportions of total reads mapping to the viral genomes. Of the sRNAs from WNV-infected C6/36 cells, 0.37% of all reads matched the WNV genome, whereas only 0.045% from infected S2 cells were WNV-specific. Likewise, the LACV infected S2 cells (0.078% virus-specific) had a proportion ≥100-fold lower than observed in infected C6/36 cells (8.39%). In contrast to the WNV-infected samples, the proportions of sRNAs matching viral genome RNA were much higher in SINV-infected cells and the difference between cell types was considerably less (11% in C6/36 cells and 4.8% in S2 cells). These findings are consistent with the 13.9% of sRNAs matching the SINV genome observed in *Ae. aegypti* four days post SINV inoculation [6]. These results may reflect differences in the replication kinetics of the viruses in each of the two cell lines. For instance, WNV infectious titers in S2 cells seven days post infection are usually 2–3 logs lower than titers in C6/36 cells five days post infection (data not shown) [29]. A relatively small proportion of small RNAs in flavivirus-infected cultured mosquito cells and mosquitoes are virus-specific as compared to mosquito infections by members of other arbovirus families. Although this has not previously been shown for bunyaviruses, it was independently determined in previous studies of flaviviruses [1], [7] (Scott *et. al.*, submitted) and alphaviruses [5], [6] and is confirmed by our current results. This could reflect a more effective mechanism of evasion of innate immunity by flaviviruses, such as sequestration of the viral replication complex in membrane-enclosed vesicles in mosquito cells as well as mammalian cells [38], [39].

Analysis of sRNA reads aligning to the various viral genomes revealed obvious differences that may be related to their biogenesis. The average length of viRNAs mapping to WNV, SINV and LACV genomes from infected S2 cells was approximately 21 nt with the majority being 20-22-mers (Figure 1). These observations are indicative of Dcr2 processing of viral RNA and are consistent with previous analyses of viRNAs from WNV infected *Cx. p. quinquefasciatus* midguts, SINV infected *Ae. aegypti* and O'nyong-nyong infected *Anopheles gambiae* [1], [5], [6]. viRNA populations generated in C6/36 cells were markedly different. The average length of sRNA reads mapping to the SINV and LACV genomes was 25.2 nt with a comparatively small proportion composed of 20–22-mers. These results are consistent with those from two *Flaviviruses*, dengue virus (DENV) and cell fusing agent virus (CFAV) [40]. The abundant of 24-28 nt long viRNAs may represent products of the piRNA pathway. piRNAs are typically derived from a positive-polarity strand in a Dcr1 and Dcr2 independent manner and are thought to control the development of reproductive tissues and the transcription of transposons [41]-[43]. Recently, what appear to be virus-derived piRNAs have been identified in *Drosophila* ovary somatic sheet cells [44]. While SINV and LACV sRNAs were not limited to a single strand, the size distributions suggest their biogenesis may have occurred through the piRNA pathway. Interestingly, the size distribution of viRNAs from WNV infected C6/36 cells was quite different from the other viruses (SINV and LACV) (Figure 1) and from DENV and CFAV [40], lacking the characteristic peak at ∼27 nt and casting doubt on the role of the piRNA pathway in their biogenesis. However, the WNV viRNAs were almost exclusively derived from the positive strand, a characteristic of piRNA biogenesis (Figure 2A). The reasons for this apparent paradox are not clear, but may be due to either technical problems or biological mechanisms. A technical explanation seems unlikely because all libraries were processed under the same conditions using identical protocols. Future experiments are required to fully examine this observation. Pre-adenylated 3′ adapters, which would have minimized our sampling of small RNA degradation products, were not utilized in these experiments [17]. Nevertheless, the paucity of 20–22 nt Dcr2-like sRNAs we observed strongly suggests that the majority of the sRNA reads mapping to the viral genomes from C6/36 cells were derived either from the piRNA pathway or cellular degradation pathways.

Examination of the polarity (positive sense vs. negative sense) of the sRNA reads mapping to the viral genomes further highlighted the differences between C6/36 and S2 cells. Whereas 16% of the likely Dcr2 generated viRNAs in WNV infected S2 cells were derived from negative-sense strand, almost no viRNAs from C6/36 cells originated from the negative-sense strand (0.1%). This may explain why WNV-derived sRNAs were not detected in C6/36 cells by Chotkowski *et. al*. as the northern blot probes used in that study were homologous to the NS1 positive sense-strand [29], and would have been unlikely to detect the extremely small proportion of negative sense sRNAs from C6/36 cells mapping to this region. Similarly, there was a 20% excess of positive-sense (vRNA) targeting in SINV infected C6/36 cells compared to S2 cells. The ratio of vRNA- to cRNA-derived viRNAs in infected S2 cells more closely resembled the ratio found in SINV infected *Ae. aegypti* [6]. Likewise, notable differences were observed in strand polarity ratios between S2 and C6/36 cells infected with LACV. In both cell types, the positive-sense (cRNA) strand was targeted: 72.5% and 84% of the viRNAs in C6/36 and S2 cells, respectively. Although this propensity for positive-sense (cRNA) LACV targeting differed from the observed positive-sense (vRNA) targeting of WNV and SINV, this discrepancy might be due to the marked differences in viral replication and gene expression mechanisms between positive- and negative-sense RNA viruses. The genomes of positive-sense RNA viruses serve as both mRNA and templates for asymmetrical replication through a negative sense RNA intermediate; however, the negative-sense RNA genomes of bunyaviruses serve as templates for both full length complementary RNA replicative intermediates and transcription of highly abundant subgenomic mRNA [45]. Furthermore, since dsRNA is undetectable by staining with a specific antibody in either LACV-infected mammalian cells [46] or mosquito cells (K. Poole-Smith, personal communication) the trigger for initiation of RNAi is unknown. Together, our data for all three viruses suggest that both replicative intermediates containing both genome-sense and anti-sense RNAs, and intra-strand secondary structures within mRNA are targeted by RNAi or other cellular nucleases. The mRNA strand bias observed in all the samples most likely reflects the proportionate abundance of mRNAs as well as use of an alternative small RNA processing pathway in C6/36 cells [40].

For both WNV and SINV in S2 cells, our findings are consistent with small RNA processing by the exogenous siRNA pathway as seen in infected mosquitoes [1], [6]. On the other hand, the increased positive-sense RNA targeting in C6/36 cells suggests that an alternate mechanism may be acting upon viral RNAs. A deep sequencing sRNA dataset was generated from WNV infected DF-1 chicken cells. Upon comparison of the WNV derived sRNAs to the C6/36 cell WNV viRNAs, it was determined that the intensity of viRNA targeting of each nucleotide of the genome was significantly correlated (Spearman r = 0.8882; p<0.0001) (data not shown). However, no correlation was observed between the S2 samples and the C6/36 (p = 0.3126) or DF-1 (p = 0.8467) samples. Since the role of RNAi in vertebrate cellular innate immunity is currently unclear and mRNA turnover pathways are conserved among metazoans, including C6/36 cells, we propose that the WNV derived sRNA populations from C6/36 cells are most likely degradation products or virus-derived piRNAs [17], [44], [47]–[49]. Further, in SINV infected C6/36 cells, the region 3′ to the subgenomic promoter on the positive-sense vRNA was intensely targeted. Were this region highly susceptible to RNAi targeting, then a similar topography would have been observed in S2 cells and *Ae. aegypti*, but this was not the case (Figure 3) [6]. A more likely explanation is that the highly abundant subgenomic transcripts were not targeted by RNAi, but rather by RNA degradation pathways or the piRNA pathway [44]. Together these results suggest that arboviruses are targeted by the antiviral exogenous siRNA pathway in *Drosophila* S2 cells, but not C6/36 mosquito cells.

The results presented in this study demonstrate that in C6/36 cells, the absence of typical siRNAs, the hallmark of RNAi mediated antiviral immunity, is not limited to WNV and is evident in infections by other diverse arboviruses, such as SINV and LACV. There are multiple steps within the antiviral RNAi response that may be responsible for the observed dysfunction. However, a recent study suggests that it may be related to lack of Dcr2 activity. Studies with cell-free lysates of C6/36 cells revealed that they are unable to process 500 bp dsRNA into 21-mers; however, complementation of C6/36 cell lysates with recombinant human Dcr restored normal dsRNA processing and the presence of detectable 21-mers [40]. Further, when C6/36 cells were co-transfected with an EGFP expression plasmid and either EGFP siRNA or EGFP long dsRNA only the siRNA was able to suppress EGFP expression (J Scott *et. al.*, submitted). These combined with our results suggest that the observed dysfunction is indeed related to lack of dicing activity and more precisely Dcr2 itself. These findings suggest that C6/36 cells may fail to accurately model important aspects of mosquito-arbovirus interactions.
